# Reversible Severe Pulmonary Hypertension after Adenotonsillectomy: A Case Report of a Child Treated at Bugando Medical Centre, Northwestern Tanzania

**DOI:** 10.1155/2016/2897320

**Published:** 2016-08-22

**Authors:** Rogatus Kabyemera, Neema Chami, Neema Kayange, Respicius Bakalemwa, Antke Zuechner, Tumaini Mhada, Gustave Buname, Adolfine Hokororo, Johannes Kataraihya

**Affiliations:** ^1^Department of Pediatrics, Bugando Medical Centre, P.O. Box 1370, Mwanza, Tanzania; ^2^Department of Pediatrics, Catholic University of Health and Allied Sciences-Bugando, P.O. Box 1464, Mwanza, Tanzania; ^3^Department of Otorhinolaryngology, Bugando Medical Centre, P.O. Box 1370, Mwanza, Tanzania; ^4^Department of Internal Medicine, Bugando Medical Centre, P.O. Box 1370, Mwanza, Tanzania; ^5^Department of Internal Medicine, Catholic University of Health and Allied Sciences-Bugando, P.O. Box 1464, Mwanza, Tanzania

## Abstract

Upper airway obstruction (UAO) due to adenotonsillar hypertrophy represents one of the rare causes of pulmonary hypertension in children. We report a case of adenotonsillar hypertrophy, managed at pediatric and otorhinolaryngology departments in Bugando Medical Centre (BMC), northwestern Tanzania, with complete remission of symptoms of pulmonary hypertension following adenotonsillectomy. A 17-month-old boy presented with difficulty breathing, dry cough, and noisy breathing since 1 year. He had facial and lower limb oedema with a pan systolic murmur at the tricuspid area, fine crepitations, and tender hepatomegaly. A grade II tonsillar hypertrophy and hypertrophied adenoids were seen on nasal and throat evaluation. A 2D-echocardiography showed grossly distended right atrium and ventricle, dilated pulmonary artery, and grade III tricuspid regurgitation. His final diagnosis was severe pulmonary hypertension with right-sided heart failure due to adenotonsillar hypertrophy. He had complete remission of cardiopulmonary symptoms after adenotonsillectomy and had normal control echocardiography six and twelve months after surgery. Children with symptoms of upper airway obstruction and cardiopulmonary involvement could benefit from routine screening for pulmonary hypertension. Adenotonsillectomy should be considered for possible complete remission of both UAO and cardiopulmonary symptoms.

## 1. Introduction

Upper airway obstruction (UAO) in children might result from different causes such as craniofacial malformations, choanal atresia, subglottic stenosis, and adenotonsillar hypertrophy (ATH) [1]. Chronic UAO can lead to hypoxaemia, hypercarbia-induced respiratory acidosis, and pulmonary vasoconstriction, which in turn may cause right ventricle (RV) dysfunction, pulmonary hypertension, and cor pulmonale [1–4]. However, cardiopulmonary complications can completely resolve following adenotonsillectomy when indicated. We hereby describe a case of pulmonary hypertension due to adenotonsillar hypertrophy which presented with symptoms of upper airway obstruction in infancy.

## 2. Case Presentation

A 17-month-old male patient was referred to our pediatric department at Bugando Medical Centre (BMC) with history of difficulty breathing since one year, excessive sweating on breastfeeding, and mouth breathing. This was accompanied with excessive noisy breathing and dry cough, with apneic spells when asleep. He also had facial and lower limbs swelling. Past two admissions were due to similar complaints where he was treated with antibiotics as a case of recurrent respiratory tract infections before referral to our hospital. His immunizations were up to date according to Tanzania Expanded Programme of Immunization (EPI).

On examination, he had facial and pitting lower limb oedema. His weight for height *Z*-score was within the median range (normal). His temperature was 36.5°C and he had respiratory rate of 48 breaths/minute and pulse rate of 120 beats/min. The oxygen saturation (SaO_2_) was 85% on room air and raised to 94% on oxygen. He did not have any visible congenital anomalies.

The apex beat was palpated at 7th intercostal space and a grade III systolic murmur was auscultated at the tricuspid area. He also had fine basal crepitations and a tender hepatomegaly, but no ascites or splenomegaly. Ear, nose, and throat evaluation revealed a high arched palate, an anterior overbite, tonsillar hypertrophy grade II (Brodsky scale), and hypertrophied adenoids.

**Figure 1:**
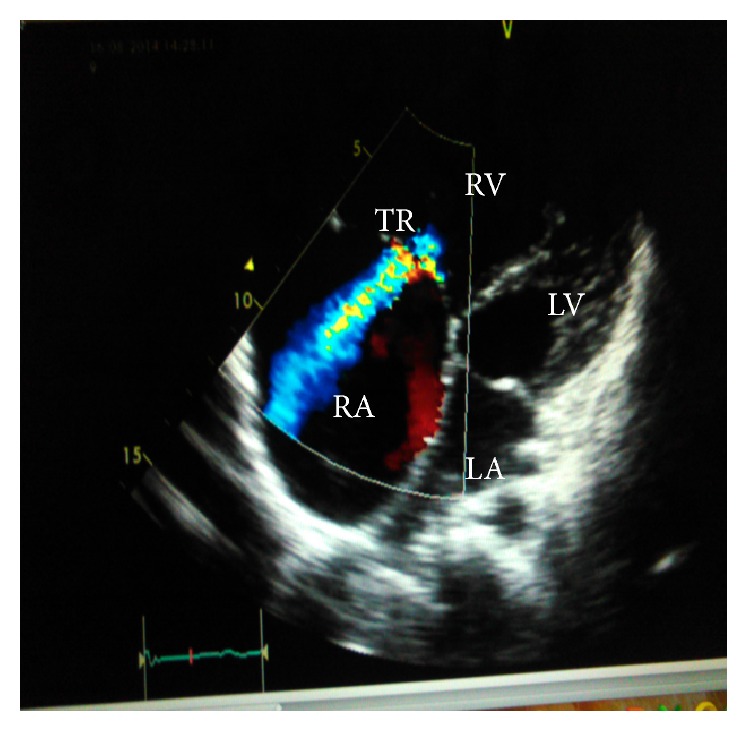
A 4-chamber view with color Doppler showing the tricuspid regurgitation (TR), enlarged right atrium (RA), enlarged right ventricle (RV), compressed left atrium (LA), and compressed left ventricle (LV) before adenotonsillectomy.

**Figure 2:**
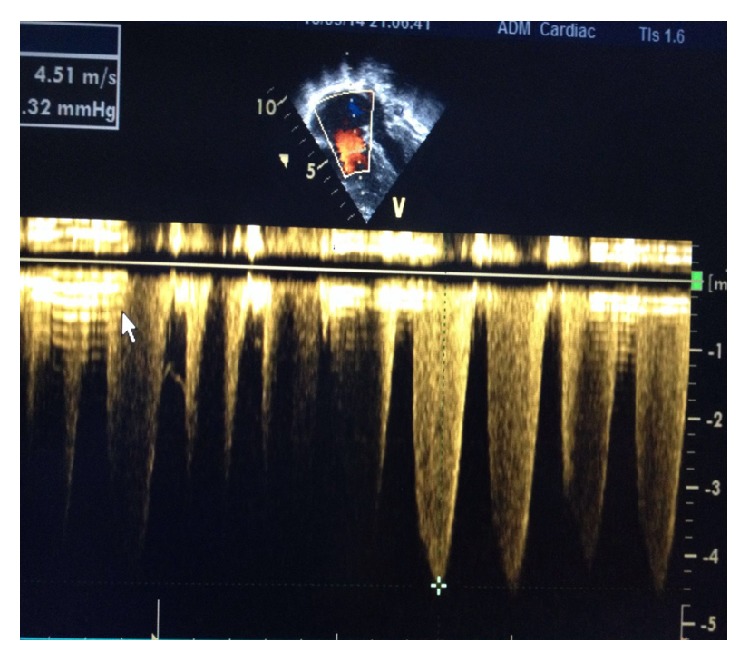
+ shows maximum velocity (4.51 m/sec) equivalent to the pressure gradient of 81 mmHg which indicates severe pulmonary hypertension before adenotonsillectomy.

Echocardiography revealed a grossly dilated right atrium and ventricle, compressed left atrium and ventricle, dilated pulmonary artery, inferior vena cava and hepatic vein, severe pulmonary hypertension (PHT), and tricuspid regurgitation grade III (Figures 1 and 2). His chest X-ray showed cardiomegaly (with the cardiothoracic ratio of 0.6) and an increased pulmonary venous vasculature. HIV rapid antibody test was nonreactive and he had lymphocytosis on the complete blood count.

He was therefore diagnosed to have pulmonary hypertension with right-sided heart failure most likely secondary to obstructive adenotonsillar hypertrophy. We started him on Furosemide (1 mg/kg 8 hourly), Aldactone (1 mg/kg 12 hourly), and oxygen therapy. Adenotonsillectomy was done 18 days later and he was discharged 2 days after surgery with normal SaO_2_ and without complications. He was thereafter followed up at our cardiac pediatric outpatient department (POPD) after every 3 months for 1 year.

**Figure 3:**
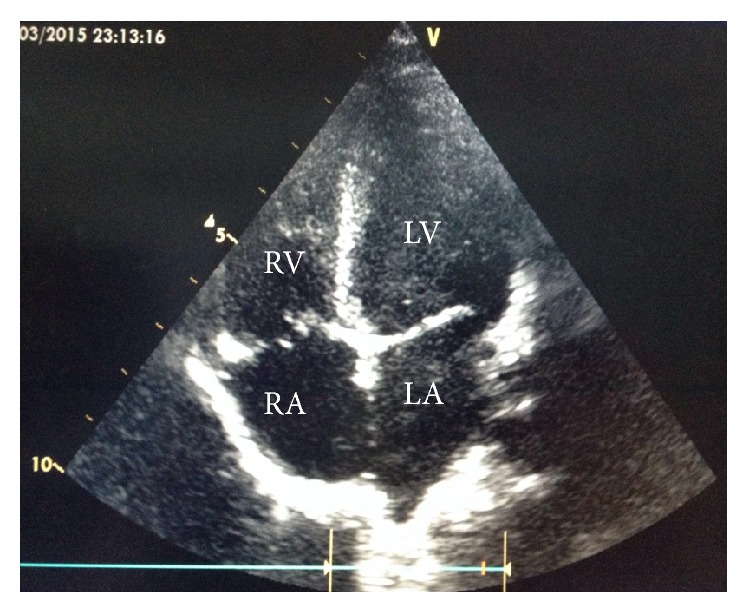
A 4-chamber view showing normal right atrium (RA), normal right ventricle (RV), normal left atrium (LA), and normal left ventricle (LV) 6 months after adenotonsillectomy.

Six and twelve months after adenotonsillectomy, echocardiography revealed normal findings indicating reversal of all cardiac alterations which were seen in the first (preoperative) and the second (six months after adenotonsillectomy, Figure 3) echocardiogram. Additionally, his obstructive nasal symptoms and signs such as snoring and mouth breathing had completely resolved. He was thereafter discharged from our cardiac pediatric outpatient department (POPD) after one year of follow-up visits.

## 3. Discussion

Severe pulmonary hypertension as a complication of adenotonsillar hypertrophy (ATH) is reported rarely in children but may be more common than previously realized. The index case report aimed to describe reversible cardiopulmonary complications of UAO in a 17-month-old child with ATH who was previously managed as a case of recurrent respiratory tract infections due to overlapping symptoms and signs. Our patient presented with recurrent history of mouth breathing, excessive snoring, and apneic spells when sleeping which are typical symptoms of UAO due to ATH [5]. This indicates the possibility of the chronic airway obstruction which may have led to persistently elevated pulmonary vascular resistance resulting in dilatation and hypertrophy of the right side of the heart and eventual right ventricular dysfunction [6]. Symptoms and signs of severe hypoxemic pulmonary hypertension, as seen in our patient, were previously reported to be sequela of chronic UAO requiring multiple admissions, antibiotic therapy, oxygen therapy, and admission to an intensive care unit [7].

We were able to confirm the diagnosis of severe PHT in our patient using the Doppler echocardiography. This investigation was described as a reliable, safe, and noninvasive alternative procedure to cardiac catheterization for routine evaluation before and after adenotonsillectomy for assessment of pulmonary arterial hypertension [5]. Though our patient had clinical signs of cardiopulmonary involvement, previous studies have demonstrated the role of routine echocardiography in clinically stable children with ATH. For instance, 65.7% of clinically normal children with adenoid hypertrophy were found to have abnormalities in the pulmonary function [6]. Another study reported that 3% of the 92 children who were scheduled for adenotonsillectomy had signs of right heart involvement on echo without clinical signs [8]. We therefore recommend that every child with adenotonsillar hypertrophy should be clinically evaluated for PHT and that the Doppler echocardiography be done in those with symptoms of right-sided heart dysfunction in settings with limited cardiac catheterization facilities before and after surgical intervention. Doppler echo should also be done first, even if cardiac catheterization laboratory is available. This may assist in identifying which patient should be prioritized for surgical intervention.

The severity of UAO symptoms depends on the size of the adenoid, tonsil, or both adenoids and tonsils [9, 10]. Surgical treatment, which was also done in our patient, has been reported to be an effective intervention in reversing the effects of UAO on the cardiovascular system [3, 11–13]. Adenotonsillectomy which was done in our patient was the definitive treatment for ATH and reversed signs of severe PHT in our patient.

In conclusion, upper airway obstruction due to adenotonsillar hypertrophy can cause severe pulmonary hypertension in children with complete remission of cardiopulmonary symptoms after adenotonsillectomy. Performing echocardiographic examination in children with adenotonsillar hypertrophy is beneficial for assessing the cardiopulmonary status of the patient and may be useful in prioritizing patients for adenotonsillectomy. Clinical evaluation for pulmonary hypertension and early referral for surgery are recommended in children with adenotonsillar hypertrophy and cardiopulmonary symptoms and signs in settings with limited resources.
